# IGSF6 is a novel biomarker to evaluate immune infiltration in mismatch repair-proficient colorectal cancer

**DOI:** 10.1038/s41598-023-47739-9

**Published:** 2023-11-21

**Authors:** Yu-ming Rong, Yu-cheng Xu, Xiao-chuan Chen, Min-er Zhong, Ying-xin Tan, Yu-fan Liang, Jing-rong Weng, Jun Liu, Xin-you Wang, Dan-dong Luo, Yi-ran Bie, Xi Chen, Jia-wei Cai, Zhao-liang Yu, Yi-feng Zou

**Affiliations:** 1 Department of VIP Region, Cancer Center of Sun Yat-Sen University, Guangzhou, Guangdong China; 2 Department of General Surgery, The Sixth Affiliated Hospital of Sun Yat-Sen University, Guangzhou, Guangdong China; 3 Guangdong Provincial Key Laboratory of Colorectal and Pelvic Floor Diseases, The Sixth Affiliated Hospital, Sun Yat-Sen University, 26 Yuancun Erheng Rd, Guangzhou, 510655 Guangdong China; 4 Department of Obstetrics and Gynecology, The Sixth Affiliated Hospital of Sun Yat-Sen University, Guangzhou, Guangdong China; 5 Department of General Surgery, Guangdong Provincial People’s Hospital, Guangdong Academy of Medical Sciences, Guangzhou, China; 6 Vascular Surgery, The First Hospital of Lanzhou University, Lanzhou, China

**Keywords:** Cancer, Immunology, Biomarkers, Diseases

## Abstract

Immunotherapy has dramatically changed the landscape of treatment for colorectal cancer (CRC), but currently lack of effective predictive biomarker, especially for tumors with mismatch repair (MMR) proficiency. The response of immunotherapy is associated with the cell–cell interactions in tumor microenvironment, encompassing processes such as cell–cell recognition, binding, and adhesion. However, the function of immunoglobulin superfamily (IGSF) genes in tumor immune microenvironment remains uncharacterized. This study quantified the immune landscape by leveraging a gene expression matrix from publicly accessible databases. The associations between IGSF6 gene expression and immune cell infiltration were assessed. The expression levels of IGSF6, CD8+ T cells, CD4+ T cells and CD68+ macrophage cells in cancer tissues from CRC patients and CRC cell lines were evaluated. IGSF6 was more highly expressed in CRC tumor tissues than adjacent normal tissues. And IGSF6 was significantly correlated with immune cell infiltration in MMR-proficient patients. Remarkably, MMR-proficient patients with high IGSF6 expression showed more sensitive to immunotherapy and chemotherapy than those with low IGSF6 expression. In summary, IGSF6 could be a novel biomarker to evaluate immune infiltration and predict therapeutic effect for MMR-proficient CRC.

## Introduction

Immunotherapy is demonstrated as a promising strategy for many types of cancers with long-term durable responses, such as for breast cancer^1^, lung cancer and melanoma^2,3^. But there are still lots of challenges in immunotherapy for colorectal cancer (CRC) patients. CRC are classified into MMR-deficient (microsatellite instability) and MMR-proficient (microsatellite stability) subtypes. Programmed death 1 (PD-1) blockade is verified as breakthrough therapy for MMR-deficient CRC, but less effective in MMR-proficient CRC^4–6^. MMR-deficient tumors are characterized by a high tumor mutational burden (TMB) and high infiltration of activated CD8+ cytotoxic T lymphocytes (CTL) and activated Th1 cells with IFN-γ production^7^. These features enable MMR-deficient tumor to be a good target for immunotherapy^8^. On the contrast, MMR-proficient CRC has been long thought to have an inactive response to immune checkpoint inhibitors. The lack of immune infiltration and low TMB in MMR-proficient CRC decrease the potential of obtaining benefits from immunotherapy, which is defined as an “immune resistant” phenotype^9^. Interestingly, about 45% of MMR-proficient tumors had a high immunoscore, which means that some of MMR-proficient tumors may be sensitive to immunotherapy^10^. Consistently, a previous clinical study reported that 27% (4/15) of MMR-proficient patients had pathological responses when treating with ipilimumab plus nivolumab, indicating that immunotherapy may be effectivity in some of MMR-proficient patients^6^. In addition, immune hot tumors with extensive immune infiltration also had better responses to chemotherapy^11,12^. However, currently lack of routinely utilise predictive biomarkers for MMR-proficient patients. As more than 90% CRC are MMR-proficient, it is an urgent clinical need to identify effective immune checkpoints for MMR-proficient patients to predict sensibility of immunotherapy.

Increasing evidences show that the tumor immune contexture, including spatial organization and density, directly influence the clinical outcome of cancer^13–16^. The immunoglobulin superfamily (IGSF) consists of the immunoglobulins (IG), T cell receptors (TR) and proteins that have the common feature of having at least one Ig-like domain^17^. It has been reported that IGSF genes are frequently overexpressed in several cancer types and plays a role in promoting cancer cell growth in the progression of cancer, such as thyroid cancer^18^ and prostate cancer^19^. Recently, a study reported that IGSF protein signatures are associated with distinct tumor immunophenotypes and clinical outcome^20^. Moreover, some studies reported the involvement of IGSF6 in the immunoregulation of atherosclerosis and inflammatory bowel disease^21,22^. Shen et al. observed a significant upregulation of CD8+ T cell infiltration in cervical cancer samples compared to normal cervical samples. Utilizing both univariate and multivariate Cox regression analyses, it was determined that CD8+ T cell infiltration was the sole independent favorable prognostic factor in cervical cancer. To further elucidate the genes associated with CD8+ T cell infiltration in cervical cancer, subsequent analyses were performed and found IGSF6 is one of the key determinants. Moreover, an elevated expression of IGSF6 is indicative of a more favorable prognostic marker^23^. However, it is unclear whether IGSF genes could be used as biomarkers in CRC. In this study, we evaluated a group of IGSF genes in CRC and found IGSF6 may be a novel prognostic biomarker for MMR-proficient CRC. We identified IGSF6 was upregulated in CRC tissues, which was correlated with the immune checkpoint genes and immune cell infiltration. Moreover, we demonstrated overexpression of IGSF6 was associated with a high density of CD8+ T cell and CD4+ T cell tumor-infiltrating lymphocytes. Importantly, MMR-proficient tumors with high IGSF6 expression showed a better response to immunotherapy and chemotherapy.

## Materials and methods

### Study design and patient selection

Six public cohorts, The Cancer Genome Atlas (TCGA) CRC cohort, GSE39582, GSE146771, GSE132465, GSE132257 and GSE144735 dataset from the UCSC Xena platform and the Gene Expression Omnibus (GEO) database, with gene expression data derived from CRC samples were evaluated retrospectively, including three bulk tumor RNA sequencing and three single cell RNA sequencing datasets. A total of 302 patients diagnosed as colon or rectal cancer and treated in the Sixth Affiliated Hospital of Sun Yat-Sen University (Guangzhou, China), 6 MMR-proficient CRC patients treated by immune checkpoint inhibitors (ICI) plus chemotherapy and 21 MMR-proficient CRC patients underwent neoadjuvant chemotherapy at the Sixth Affiliated Hospital of Sun Yat-Sen University (Guangzhou, China), were retrospectively studied. Patients with infectious diseases, autoimmune diseases, or multiple primary cancers were excluded. The study was conducted in accordance with the Declaration of Helsinki and approved by the Institutional Review Board (IRB) of the Sixth Affiliated Hospital, Sun Yat-sen University (approval number: 2021ZSLYEC-099).

### Cell lines

All cell lines, including human macrophage cell line, and colorectal cancer cell lines, were sourced from the American Type Culture Collection (ATCC) in Manassas, VA. The cells were cultivated in either DMEM or RPMI media, both supplied by Invitrogen, enriched with 10% fetal bovine serum (FBS) from MIKX, China and 1% penicillin–streptomycin solution from Gibco. Cultures were maintained at a consistent temperature of 37 °C within a 5% CO_2_ incubator.

### Western blot analysis

Cellular extracts were meticulously harvested using RIPA lysis buffer, comprised of a precise composition (50 mM Tris–HCl at pH 7.4, 1 mM EDTA, 0.25% deoxycholic acid disodium salt, 1% NP40, 150 mM NaCl, 0.1% SDS). This was further augmented with protease inhibitors sourced from TargetMol Chemicals (Catalog No. C0001), USA, alongside PMSF. Protein quantification was facilitated through the BCA assay, post which 30 μg of the cellular lysates were electrophoresed via SDS/PAGE alongside a prestained protein marker (M221, GenStar, Beijing, China). This fractionated protein sample was subsequently transferred onto PVDF membranes, courtesy of BioRad, USA. Post a 2-h blocking phase, the membranes underwent overnight incubation at 4 °C with anti-IGSF6 (Santa Cruz, sc-377053, 1:500). This was succeeded by a triple rinse with TBST. Further incubation ensued with a secondary antibody, conjugated with horseradish peroxidase, for an hour at ambient conditions. The culmination of this rigorous process was the illumination of protein-specific bands, achieved using the chemiluminescence reagent (ECL, procured from Millipore, USA).

### Immunofluorescence

Sections of 5 μm thickness were obtained from CRC tumors. Deparaffinization of slides was done with xylene followed by rehydration in histological-grade ethanol and fixation with 3% hydrogen peroxide in methanol, before antigen retrieval using pH 6.0 citrate buffer or pH 9.0 EDTA buffer. Then the slides were incubated with the primary anti-IGSF6 (Santa Cruz, sc-377053, 1:200), anti-CD4 (ZSGB-Bio, ZA-0519), anti-CD8 (ZSGB-Bio, ZA-0508), anti-CD68 (ZSGB-Bio, ZM-0060) and anti-CEA (ZSGB-Bio, ZA-0063) overnight at 4 °C. Slides were scanned and digitalized with and visualized with CASEVIEWER software.

### Immunohistochemistry staining

Immunohistochemistry (IHC) and hematoxylin and eosin (H&E) staining were performed using standard immunoperoxidase staining on CRC tissue sections of 5 μm thickness from resected tumors. Sections were stained against anti-IGSF6 (Santa Cruz, sc-377053, 1:200), anti-CD4 (ZSGB-Bio, ZA-0519), anti-CD8 (ZSGB-Bio, ZA-0508) and anti-CD68 (ZSGB-Bio, ZM-0060). Paraffin sections were deparaffinized with xylene in the stainer and then underwent heat-mediated antigen retrieval with pH 9.0 EDTA buffer. Then the slides were incubated with the primary antibody overnight at 4 °C, and the sections were stained with diaminobenzidine. Sections were counterstained with hematoxylin, dehydrated and mounted with coverslips. Slides were scanned and digitalized with the TEKSQRAY image analysis system and visualized with ImageViewerG software. A board-certified pathologist evaluated the staining digitally to ensure the appropriate quality of tumor tissue.

### Evaluation of immunohistochemical and immunofluorescence analysis

Immunoreactivity for IHC and immunofluorescence staining was evaluated by a semiquantitative method, as described previously. Each TMA spot was assigned an intensity score from 1 to 4 (1, 2, 3, or 4) by two trained researchers. The percentage of positive tumor cells divided by the total number of tumor cells was assigned using 1, 2, 3, and 4. IHC and immunofluorescence scores were determined by the intensity score and the proportion of area positively stained tumor cells. A final score was determined as the average of two cores from the same representative tumor area.

### Statistical analysis

All statistical analyses were accomplished by R software V.4.2.1 (http://www.r-project.org) and Graphpad Prism 9 software. Data were presented as the mean ± SD, unless otherwise stated. Statistical significance between two groups was evaluated by two-tailed Student’s t-test. Statistical significance was considered at *p* < 0.05.

### Ethics approval and consent to participate

This study was approved by the Institutional Review Board (IRB) of The Sixth Affiliated Hospital of Sun Yat-sen University. This is a retrospective trial from public datasets with demonstrated minimal risk and we petition BPG for waiver of ethics consent.

## Results

### IGSF6 exhibits strong association with immune infiltration in colorectal cancer and could be a novel biomarker to evaluate immune infiltration

**Figure 1:**
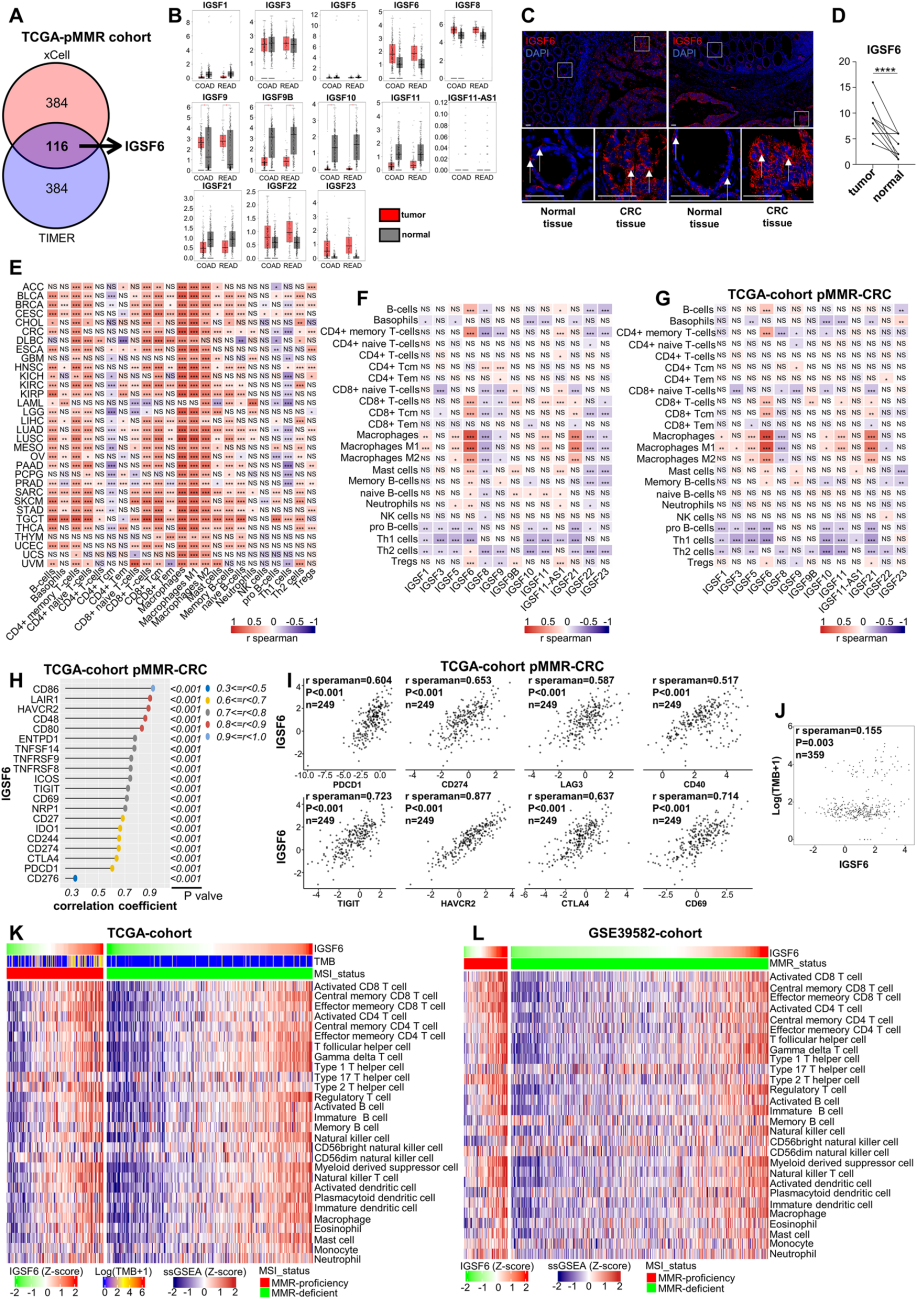
IGSF6 exhibits strong association with immune infiltration in colorectal cancer and could be a novel biomarker to evaluate immune infiltration. (**A**) In the TCGA database, a comprehensive analysis of the correlation between all genes and CD8+ T cell infiltration in pMMR colorectal cancer was performed using TIMER and Xcell algorithms. Among the top 500 genes, IGSF6 emerged as a highly ranked candidate. (**B**) Expression of IGSF in colorectal cancer from the GEPIA database. (**C**) Representative immunofluorescence images of IGSF6 expression in CRC and adjacent normal tissues from the patient. (**D**) Quantification of the score for IGSF6 level in tumor tissue versus adjacent tissue assessed by immunofluorescence assay. *p < 0.05, ***p < 0.001, ****p < 0.0001. p values were determined by paired-*t* test. (**E**) Correlation between immune cell infiltration and IGSF6 in pan-cancer, *p < 0.05, **p < 0.01, ***p < 0.001. (**F**) Correlation between immune cell infiltration and IGSF in CRC patients from TCGA database, n = 383. (**G**) Correlation between immune cell infiltration and IGSF in MMR-proficient CRC patients, n = 249. (**H**,**I**) Correlation between immune check points and IGSF6 expression in MMR-proficient patients by Spearman’s correlation coefficient. n = 249. (**J**) Correlation between TMB and IGSF6 expression in CRC patients by Spearman’s correlation coefficient, n = 359. (**K**,**L**) Unsupervised hierarchical clustering of colorectal tumors using ssGSEA scores for immune signatures identifies increasing levels of immune infiltrates. (**J**) from TCGA database, n = 367, (**K**) from GSE39582 database, n = 519.

In this study, we sought to identify biomarkers for assessing immune infiltration and predicting immunotherapy response, which is crucial for determining the efficacy of clinical treatment. To this end, we first explored the correlation between the expression of all genes and CD8+ T cell infiltration in MMR-proficient CRC patients from The Cancer Genome Atlas (TCGA) CRC cohort, utilizing TIMER and Xcell algorithms. We specifically focused on the top 500 genes according to the size of the correlation coefficient. Upon intersecting these genes, IGSF6 emerged as a prominent candidate (Fig. 1A). Moreover, among the IGSF family, only IGSF6 can be found in these genes. To characterize the potential function of IGSF genes in CRC, we next investigated the expression of IGSF genes in CRC tumor tissue and adjuvant tissue from Gene Expression Profiling Interactive Analysis (GEPIA, cancer-pku.cn). Interestingly, IGSF6 was significative up-regulated in both colon and rectal tumor versus normal tissue (Fig. 1B). To confirm the findings in public database, we detected IGSF6 expression in 16 pairs of CRC tissues and adjacent normal colorectum tissues. Unexpectedly, IGSF6 levels are highly expressed in tumor tissues as compared with adjacent normal tissues (*p* < 0.0001, Fig. 1C,D). Since IGSF genes play a central role in cell–cell recognition as cell surface receptors, these results suggest that IGSF6 may be a neoantigen generated during carcinogenesis and involving in immune infiltration.

The tumor immune microenvironment emerges as a pivotal determinant in tumor evolution and influences the efficacy of immunotherapy. However, the scientific community is currently on a quest, as an effective biomarker to gauge immune infiltration and assess the efficacy of immunotherapy remains elusive. To evaluate IGSF6 can be used as a biomarker for immune infiltration, we assessed the association between IGSF6 expression and tumor-infiltrating lymphocytes in TCGA datasets. The results showed that the expression of IGSF6 was positive correlated with tumor-infiltrating lymphocytes in various cancers, especially for CRC (Fig. 1E). Furthermore, only IGSF6, among IGSF family genes, showed a strong positive correlation with tumor-infiltrating lymphocytes in CRC patients, including MMR-deficient and MMR-proficient tumors (Fig. 1F). Interestingly, a strong positive correlation between IGSF6 expression and tumor-infiltrating lymphocytes can be found in MMR-proficient tumors, indicating that IGSF6 could be an immunotherapy biomarker for MMR-proficient CRC patients (Fig. 1G). What’s attractive, IGSF6 was strongly positive correlated with immune checkpoints in MMR-proficient CRC, such as PD-1, PD-L1, CTLA-4, LAG3 and TIGIT (Fig. 1H,I). Moreover, expression of IGSF6 correlated with tumor-infiltrating lymphocytes were confirmed in GSE39582 dataset (Supplementary Fig. 1), which supported that IGSF6 could be a potential biomarker to improve clinical applications of current immunotherapies.

MMR-deficient tumors which have highly tumor mutational burden (TMB), for accumulation of insertions–deletions (indels) which given rise to more neoantigens, are more beneficial from immunotherapy^24–28^. Therefore, we evaluated the association between IGSF6 expression and TMB in CRC, and found a positive correlation between IGSF6 genes and TMB (Fig. 1J). Moreover, we found that IGSF6 manifests a favorable expression trajectory in the MMR-proficient as compared to MMR-deficient in CRC (Supplementary Fig. 2), which suggested the potential role of IGSF6 as a potential biomarker of cancer therapy in MMR-proficient CRC.

Furthermore, we investigated the relationship between IGSF6 expression and immune cell components in tumor microenvironment. We calculated single-sample gene set enrichment analysis (ssGSEA) scores for tumor samples using immune signatures. Given that MMR-deficient tumors have a distinct immunologic profile, we performed unsupervised hierarchical clustering on the MMR-proficient tumors (Fig. 1K). We found high IGSF6 expression tumors were associated with extensive immune infiltration, which was confirmed in another CRC dataset (Fig. 1L).

**Figure 2:**
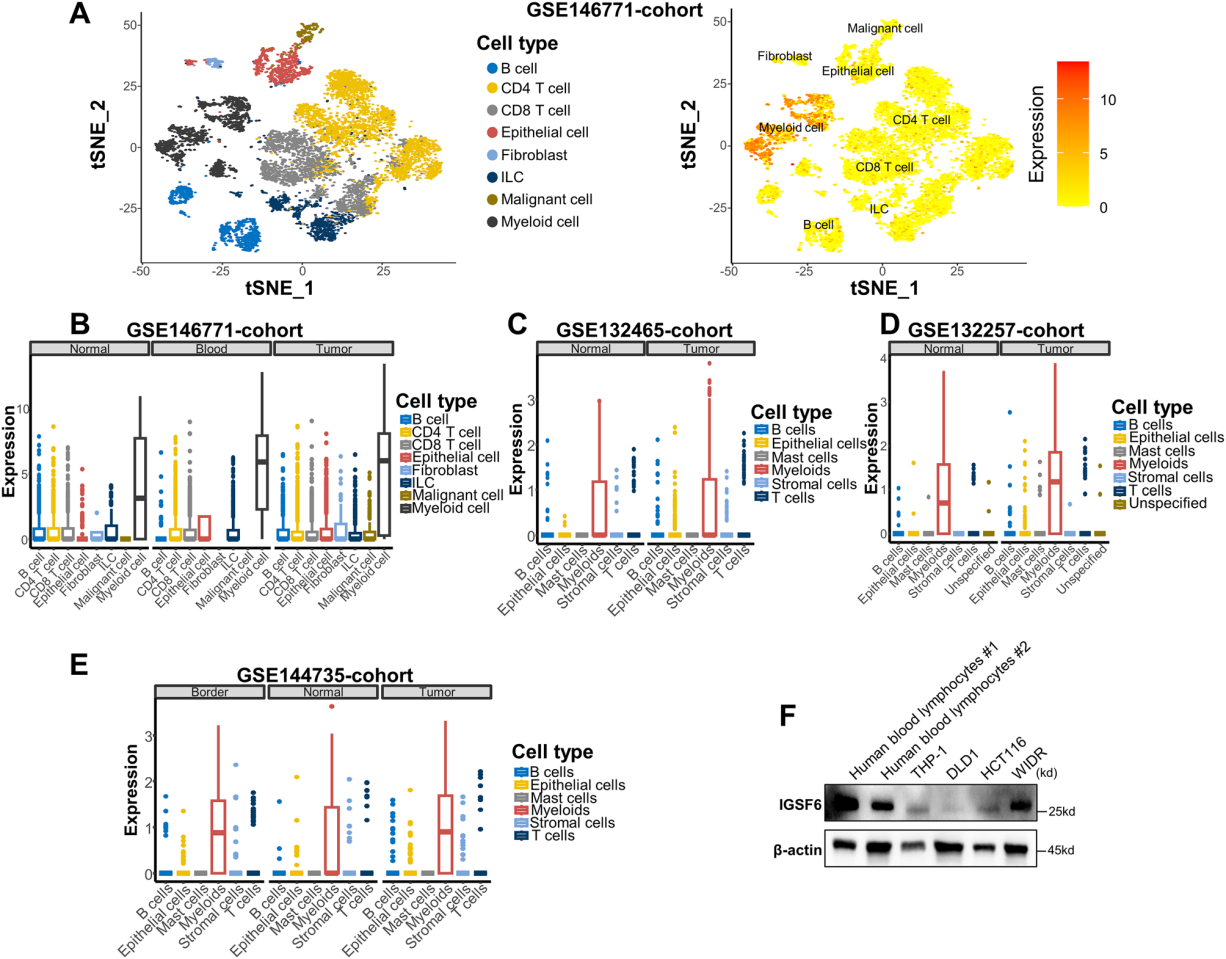
IGSF6 is highly expressed in macrophage cell. (**A**) t-SNE plot showing 8 cell type clusters (left) and expression of IGSF6 (right) in Smart-seq2 scRNA-seq GSE146771-cohort. (**B**–**E**) Expression of IGSF6 (TPM) in cell type subpopulations from GSE146771-cohort (**B**), GSE132465-cohort (**C**), GSE132257-cohort (**D**) and GSE144735-cohort (**E**). (**F**) Western blot analysis for IGSF6 in cell lines and Human blood lymphocytes from donors. β-Actin was used as a loading control. The membrane probed for IGSF6 was cropped at approximately 45 kd and 10 kd; the membrane probed for β-actin was initially cropped around 90 kd and 15 kd. Original blots are presented in Supplementary Fig. 1E.

By analysis published single-cell RNA-Seq (scRNA-Seq) database in CRC, we further investigated the transcriptional information of individual cells, shedding light on the transcriptional landscape of malignant colorectal lesions (Fig. 2A). The definition of cluster identities was ascertained based on the expression profiles of recognized marker genes. The analysis delineated the cells into eight distinct lineages. Beyond the malignant cells, other seven immune cell populations were also unveiled: B cells, CD4+ T cells, CD8+ T cells, Epithelial cells, Fibroblasts, ILCs, and Myeloid cells, thus providing a holistic view of the cellular landscape under investigation. We discerned that IGSF6 consistently manifested elevated expression levels in Myeloid cells (Fig. 2A), irrespective of their residency in tumoral or normal tissue landscapes (Fig. 2B). Additionally, these data were validated in the following datasets: GSE132465-cohort (Fig. 2C), GSE132257-cohort (Fig. 2D), and GSE144735-cohort (Fig. 2E). Next, we detected IGSF6 expression in a selection of CRC cell lines, THP-1, and lymphocytes derived from human blood donors with western blot analysis. Strikingly, our experimental data indicate that IGSF6 exhibits the strongest expression in human lymphocytes while varies expression in the THP-1 and colorectal cancer cell lines, underscoring its potential significance in these cellular environments (Fig. 2F).

**Figure 3:**
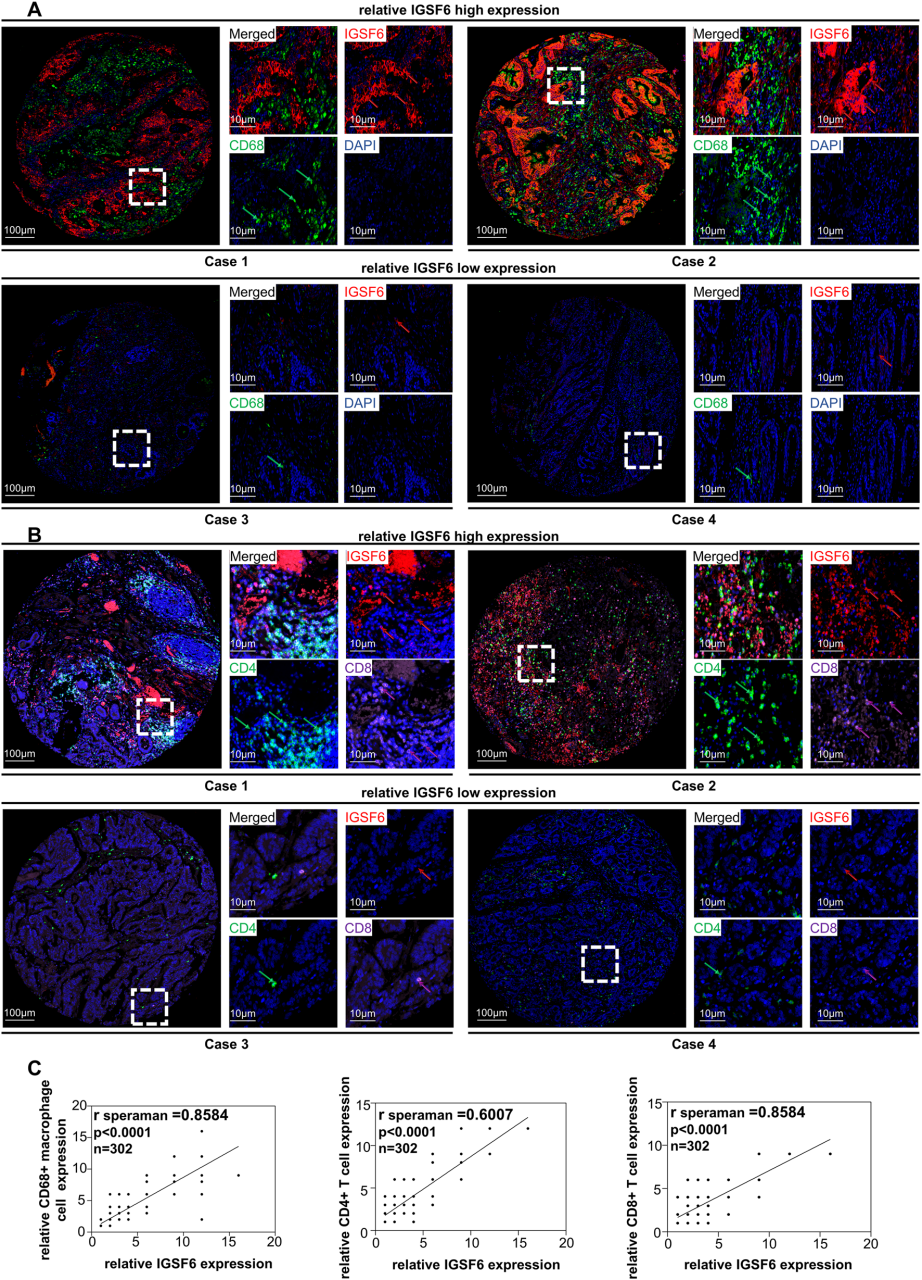
IGSF6 is highly correlated with immune infiltration in CRC tissues. (**A**) Representative immunofluorescence images of IGSF6 and CD68+ macrophage cell expression in CRC tissues from the patients, red arrowheads for IGSF6, green arrowheads for CD68+ macrophage cell (**B**) Representative immunofluorescence images of IGSF6, CD4+ T cell and CD8+ T cell expression in CRC tissues from the patients, red arrowheads for IGSF6, green arrowheads for CD4+ T cell, purple arrowheads for CD8+ T cell. (**C**) Correlation between CD4+ T cell, CD8+ T cell, CD68+ macrophage cell and IGSF6 in CRC patients by Spearman’s correlation coefficient, n = 302.

We further examined tissue microarrays of colorectal cancer specimens from a cohort of 302 patients using multiplex immunofluorescence staining. Remarkably, the experimental outcomes were consistent with the aforementioned results, revealing a high expression of IGSF6 associated with CD68+ macrophages (Fig. 3A,C). Furthermore, we constructed a tissue microarray on colon or rectal tumor tissues containing a large cohort of CRC patients (n = 302) and detected the expression of IGSF6, CD4+ and CD8+ T cell with multiple immunofluorescences. Consistent with previous studies^6^, the infiltrated CD4+ and CD8+ T lymphocytes were different in various cases of CRC tissues, which was highly correlated with tumor response to treatment (Fig. 3B). Interestingly, multiple immunofluorescences analysis indicated a strong positive correlation between IGSF6 expression and tumor-infiltrating lymphocytes, including CD4+ and CD8+ T cell, in CRC tumors (Fig. 3B,C).

### High IGSF6 expression is associated benefit from immunotherapy and chemotherapy

**Figure 4:**
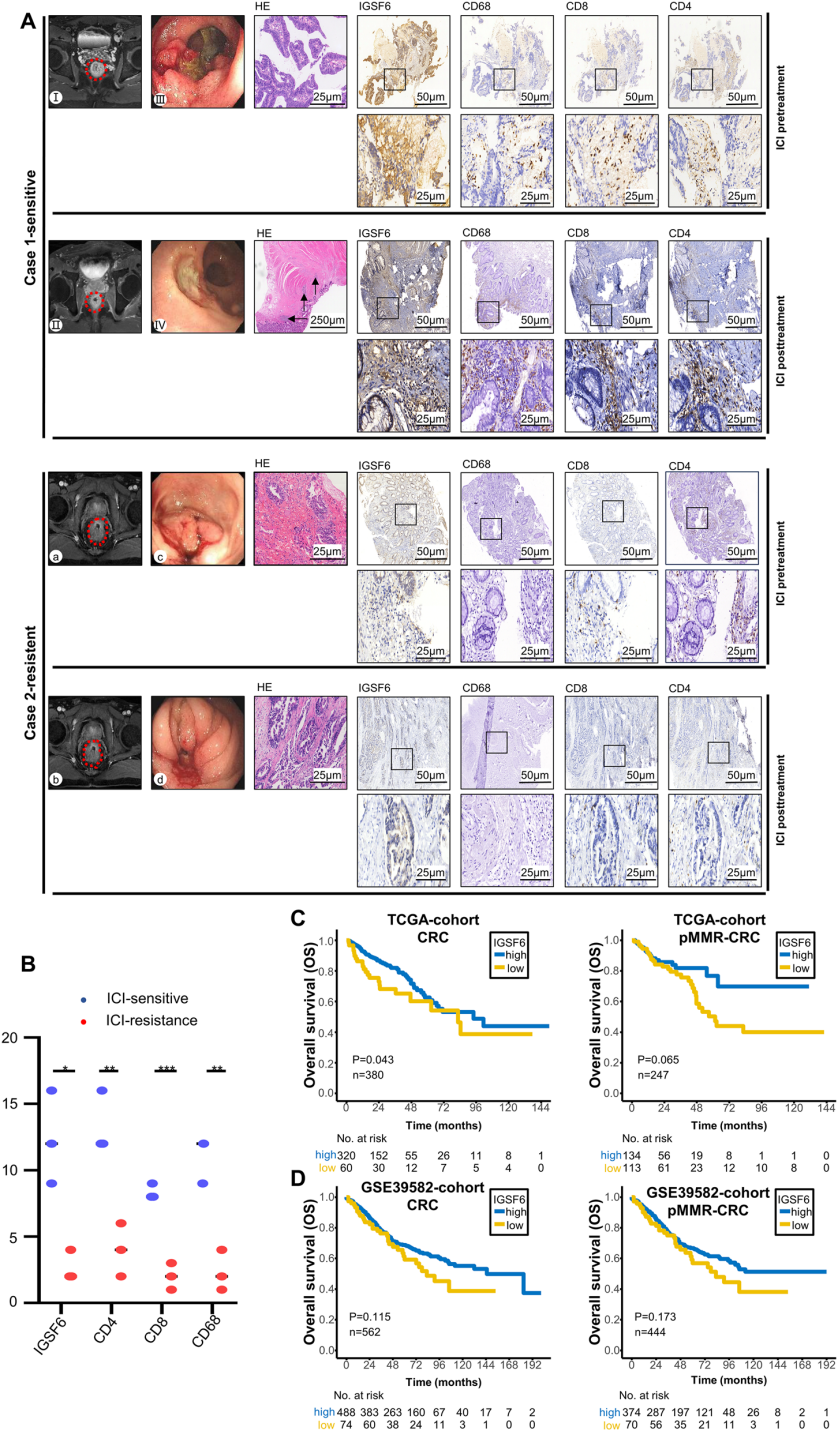
High IGSF6 expression is benefit from immunotherapy. (**A**) Radiological and pathological response to FOLFOX plus sintilimab in a pMMR patient with stage T3N0M0 (Case 1) and FOLFOX plus sintilimab in a pMMR patient with stage T3N1M0 (Case 2). Radiographic imaging shows the tumor in rectum (I, a) at initial diagnoses. A notable tumor regression could be seen in primary tumor from Case 1 (II). But there was no response in Case 2 after ICI (b). Primary tumor was observed using colonoscopy (III, c) at initial diagnosis and after ICI treatment (IV, d). H&E staining shows primary tumor at initial diagnosis and pathological response after ICI treatment. Fibrosis and an infiltration with viable density of many lymphocytes (arrowheads) can be found, which cannot be found in case 2. IHC staining showed CD4+ T cells, CD8+ T cells, CD68+ macrophage cells and IGSF6 expression with pretreatment tumor samples and posttreatment tumor tissues. (**B**) Quantification of the score for CD4+ T cells, CD8+ T cells, CD68+ macrophage cells and IGSF6 staining before treatment in CRC tissue from immunotherapy sensitive versus immunotherapy resistance assessed by IHC assay, n = 6. (**C**,**D**) OS curve of patients with high IGSF6 and low IGSF6 group in TCGA database (**C**) and GSE39582 database (**D**).

As IGSF6 expression was associated with immune infiltration, we next investigated whether IGSF6 could be used as a biomarker to predict therapeutic effect for CRC. A total of 6 MMR-proficient CRC patients treated with immunotherapy were studied (Table 1). After systematic ICI treatment, the patients received staged or simultaneous complete surgical resection for primary tumor. A thorough pathological examination of the resected tumors before and after ICI was conducted. Immuno-sensitive MMR-proficient CRC showed a sharp tumor burden reduction, massive necrosis tissue and lymphocytes infiltration after ICI treatment, such as Case 1 (Fig. 4A). On the contrary, there was no response or even bad response in immuno-resistance tumors, such as Case 2 (Fig. 4A). We next detected IGSF6 expression, CD4+ T cell, CD8+ T cell and CD68+ macrophage cell from pathological tumor specimens with IHC. Interestingly, high IGSF6 expression were observed in resected specimens in immuno-sensitive tumors, while with high CD4+, CD8+ T cell and CD68+ macrophage cell infiltration (Fig. 4A). As expected, IGSF6 levels are more highly expressed in immuno-sensitive tumors than immuno-resistance tumors (Fig. 4B). To further clarify whether the IGSF6 could predict benefit in CRC patients, we focused on the clinical outcome in the IGSF6^high^ and IGSF6^low^ groups. The OS of CRC patients with IGSF6 high expression was better than those with IGSF6 low expression in TCGA cohorts, especially for MMR-proficient patients (Fig. 4C). The same tendency can also be found in GSE39582 cohorts (Fig. 4D).

**Table 1:** Patient characteristics of 6 MMR-proficient CRC patients treated with ICI.

Characteristics	Patient 1	Patient 2	Patient 3	Patient 4	Patient 5	Patient 6
Primary tumor site	Rectum	Rectum	Rectum	Rectum	Rectum	Colon
Stage	T2N0M0	T3N0M0	T3N0M0	T3N1M0	T4aN1aM0	T3N0M0
Metastatic site	NO	NO	NO	NO	NO	NO
Treatment before ICI						
Systematic (response^¶^)	None	FOLFOX + radiotherapy (PR)	FOLFOX (PR)	None	FOLFOX (PD)	XELOX (PD)
Surgery	None	None	None	None	Hartmann	Right hemicolectomy
ICI combined treatment						
Regimen	Sintilimab + Avastin + FOLFOX	FOLFOX + sintilimab	FOLFOX + sintilimab	sintilimab + FOLFOX	Camrelizumab	Nivolumab
Systematic (response^¶^)	PR	PR	PR	PD	PD	PD
Radiological response	PR	PR	PR	PD	Ξ	Ξ
Surgical treatment (after ICI)	Dixon	TaTME	Dixon	Dixon	Ξ	Ξ
Pathological response						
Primary tumor	PR	PR	PR	PD	Ξ	Ξ
Reginal lymph nodes	PR	PR	PR	PD	Ξ	Ξ
Postoperative treatment	None	None	FOLFOX	FOLFOX	Camrelizumab	Nivolumab
TRG score (NCCN Guidelines)	1	1	1	2	Ξ	Ξ

^¶^Assessed by the Response Evaluation Criteria in Solid Tumors 1.1 criteria.

^ξ^Colectomy was conducted before ICI combined treatment.

*FOLFOX* fluorouracil + oxaliplatin, *XELOX* capecitabine + oxaliplatin, *ICI* immune checkpoint inhibitor, *pMMR* mismatch repair (MMR) proficient, *dMMR* mismatch-repair (MMR) deficient, *PD* progressive disease, *PR* partial response.

**Figure 5:**
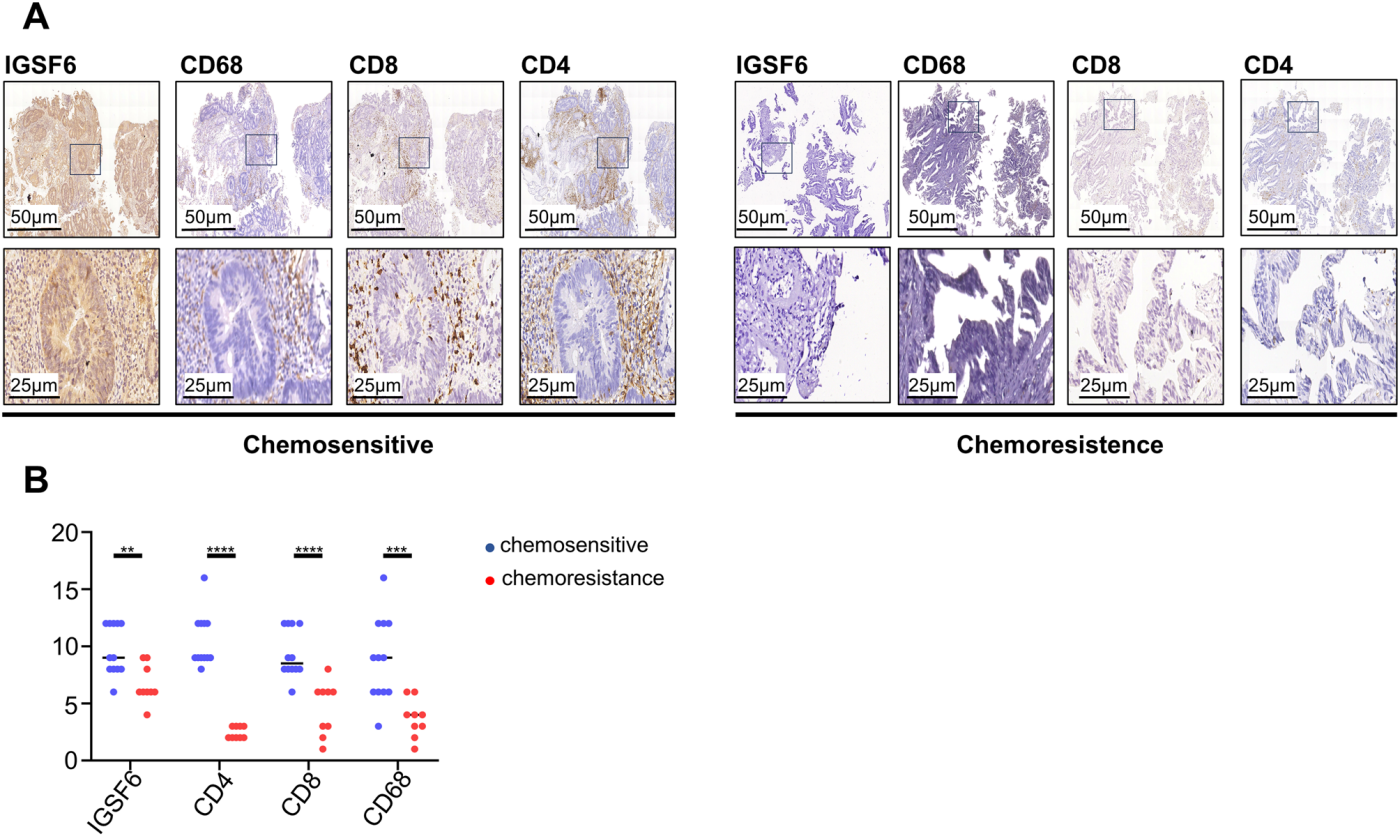
High IGSF6 expression is benefit from chemotherapy. (**A**) Representative micrographs of CD4+ T cells, CD8+ T cells, CD68+ macrophage cells and IGSF6 staining from endoscopic pathological tumor specimens before pMMR patients underwent chemotherapy, n = 21. (**B**) Quantification of the score for CD4+ T cells, CD8+ T cells, CD68+ macrophage cells and IGSF6 staining from chemosensitive CRC versus chemoresistance CRC by IHC assay, n = 21. pMMR, mismatch repair (MMR) proficiency; dMMR, mismatch repair (MMR) deficient. *p < 0.05, ***p < 0.001, ****p < 0.0001.

For the majority of MMR-proficient CRC patients, chemotherapy persists as a prevalent and efficacious therapeutic option. To further explore the relationship between IGSF6 and patients’ response to chemotherapy, we collected the fresh tissues of chemosensitive and chemoresistance MMR-proficient CRC patients before treatment (Table 2). As expected, IGSF6 levels are more highly expressed in tumor tissues from chemosensitive patients than from chemoresistance patients (Fig. 5A,B). There were more tumor-infiltrating immune cells in tumor tissues of the IGSF6^high^ group than in those of the IGSF6^low^ group, indicating that MMR-proficient CRC tissues with high levels of IGSF6 had a tumor microenvironment with an activated adaptive immune phenotype (Fig. 5A,B).

**Table 2:** Patient characteristics of 21 MMR-proficient CRC patients treated with chemotherapy.

	TRG	Treatment	Stage	Primary tumor site
Patient 1	0	FOLFOX	T3N1aM0	Rectum
Patient 2	0	FOLFOX	T4aN2aM0	Rectum
Patient 3	0	FOLFOX	T3N0M0	Rectum
Patient 4	3	FOLFOX	T3N1aM0	Rectum
Patient 5	0	FOLFOX	T4aN2aM1	Rectum
Patient 6	0	FOLFOX	T3N2aM0	Rectum
Patient 7	0	UNKNOWED	T4aN2bM0	Rectum
Patient 8	0	Avastin + FOLFOXIRI	T3N2aM1	Rectum
Patient 9	3	FOLFOXIRI	T3N1aM0	Rectum
Patient 10	3	FOLFOX	T3N1bM0	Rectum
Patient 11	0	FOLFOX	T3N2aM0	Rectum
Patient 12	3	FOLFOX	T3N2bM0	Rectum
Patient 13	3	FOLFOXIRI	T3N2bM0	Rectum
Patient 14	0	FOLFOXIRI	T3N2aM0	Rectum
Patient 15	3	FOLFOX	T3N1bM0	Rectum
Patient 16	3	FOLFOX	T3N1aM0	Rectum
Patient 17	3	XELOX	T4aN2bM1	Rectum
Patient 18	0	FOLFOXIRI	T3N1aM0	Rectum
Patient 19	3	FOLFOX	T3N0M0	Rectum
Patient 20	0	FOLFOX	T3N2bM0	Rectum
Patient 21	0	FOLFOXIRI	T3N2aM0	Rectum

*FOLFOX* fluorouracil + oxaliplatin, *XELOX* capecitabine + oxaliplatin, *FOLFOXIRI* fluorouracil + oxaliplatin + Irinotecan.

## Discussion

CRC is one of the most common cancer types in the world due to its high prevalence^29^. Immunotherapy has shown as an effectively therapeutic strategy for many types of cancers, but its clinical application in CRC only for MMR-deficient patients. A large amount of MMR-proficient tumors had shown a high proportion of inter-tumoral tumor-infiltrating lymphocytes as “hot tumors”, which may be sensitive to immunotherapy^11,12^. Biomarkers, including CEA, CA199, and CA125, in clinical practice are significance for tumor cell proliferation and tumor recurrence. But lack of effectively biomarker which can predict immune infiltration. Hence, the identification of novel predictive markers is essential to select MMR-proficient tumors that would respond to immunotherapy.

Recent studies point out the importance of the tumor immune contexture for the prognosis of patients with CRC^13–16^. Immunotherapy targeting immune checkpoints (such as PD1/PD-L1) has become an approved treatment option for patients with CRC with mismatch repair deficiency or high microsatellite instability^30^. Interestingly, about 45% of MMR-proficient and 65% of MMR-deficient CRC had a high immunoscore, while 55% of MMR-proficient and 35% of MMR-deficient CRC had a low immunoscore, suggesting that some of the MMR-deficient tumors are unable to mount an antitumor response while the reverse may apply for some of the MMR-proficient population^10^. Indeed, the 27% pathological response in MMR-proficient early-stage CRC treated with neoadjuvant ipilimumab plus nivolumab provide further support that MMR-proficient CRC is not an immune desert and can be targeted with immunotherapy^6^. Apart from MMR-deficiency and MMR-proficiency, we speculate that IGSF6 gene is another potential biomarker for responsiveness to immunotherapy due to its significant association with TMB, which is an effective indicator for response prediction to ICI. Our study found that IGSF6 may be a new biomarker which may indicate the infiltration of immune cells.

A number of inhibitory immunoreceptors have been identified and studied in cancer in past decades, including but not limited to PD-1, PD-L1, CTLA-4, LAG3, HAVCR2, TIGIT, CD69 and CD40. They are viewed as “immune checkpoints” referring to molecules that act as gatekeepers of immune responses. In the evolutionary process, immune checkpoints have co-evolved with stimulatory immunoreceptors and appear as early as in fish^31^. These receptors often use monotyrosine signaling motifs, such as immunoreceptor tyrosine-based inhibitory motif (ITIM) and immunoreceptor tyrosine-based switch motif (ITSM), to deliver inhibitory signals. As surface molecules, their activity can be easily inhibited by blocking antibodies that prevent ligand-receptor engagement. Immune checkpoint blockade therapy often leads to more durable response than chemo or targeted therapies, perhaps reflecting the memory feature of the immune system. The major bottleneck of immune checkpoint blockade therapy is its low response rate in some cancers, with a range of 10–30%^32^. For some major cancer types such as MMR-proficient CRC, anti-PD-1/PD-L1 therapy shows nearly no effect^24^. In this study, we identified a crucial involvement of IGSF6 in CRC promotion immune cells. IGSF6 expression was upregulated in CRC tissues and high expression of IGSF6 correlated with an active immune microenvironment and a favorable prognosis and furthermore it may facilitate immune mediated tumor killing. In the tapestry of MMR-proficient tumors, heightened IGSF6 expression is predominantly anchored in the immune milieu, notably emanating from infiltrating macrophages and distinct lymphocyte subsets. This surge in IGSF6 emerges as a potential biomarker for immune infiltration.

In conclusion, we elucidated that IGSF6^high^ expression as an immune related biomarker in MMR-proficient CRC, so as to facilitate immune mediated tumor killing. IGSF6^high^ expression was an independent predictor of better prognosis, and these results may provide insight into effective strategies for therapy in MMR-proficient CRC.

### Supplementary Information

Supplementary Figures.

## Data Availability

The Cancer Genome Atlas (TCGA) pan-cancer data were obtained from UCSC Xena (https://xenabrowser.net/datapages/?cohort=TCGA%20Pan-Cancer%20 (PANCAN)), including gene expression RNAseq (https://xenabrowser.net/datapages/?dataset=tcga_RSEM_gene_fpkm&host=https%3A%2F%2Ftoil.xenahubs.net&removeHub=https%3A%2F%2Fxena.treehouse.gi.ucsc.edu%3A443), phenotype—sample type and primary disease (https://xenabrowser.net/datapages/?dataset=TCGA_phenotype_denseDataOnlyDownload.tsv&host=https%3A%2F%2Fpancanatlas.xenahubs.net&removeHub=https%3A%2F%2Fxena.treehouse.gi.ucsc.edu%3A443) and phenotype—Curated clinical data (https://xenabrowser.net/datapages/?dataset=Survival_SupplementalTable_S1_20171025_xena_sp&host=https%3A%2F%2Fpancanatlas.xenahubs.net&removeHub=https%3A%2F%2Fxena.treehouse.gi.ucsc.edu%3A443). TMB data in Colorectal Adenocarcinoma (TCGA, PanCancer Atlas) were obtained from cBioPortal (https://www.cbioportal.org/study/clinicalData?id=coadread_tcga). MSI status data of The Cancer Genome Atlas (TCGA)-COAD and TCGA-READ datasets from the Genomic Data Commons were obtained from using the R package “TCGAbiolinks”. Other RNA-seq data reported in this article has been deposited in NCBI’s Gene Expression Omnibus (GEO) and are accessible through GEO Series accession number GSE39582. Single cell RNA sequencing (scRNA-seq) data of colorectal cancer reported in this article has been deposited in NCBI’s Gene Expression Omnibus (GEO) and are accessible through GEO Series accession number GSE132465, GSE146771, GSE132257, GSE144735.
