# Supplementary material for: IGSF6 is a novel biomarker to evaluate immune infiltration in mismatch repair-proficient colorectal cancer
Source: Sci Rep. 2023 Nov 21;13:20368. doi: 10.1038/s41598-023-47739-9 (PMC10663589; doi:10.1038/s41598-023-47739-9)
Supplement: Supplementary file 1 — Supplementary Figures. [file 41598_2023_47739_MOESM1_ESM.pdf]

Supplementary Figure 1

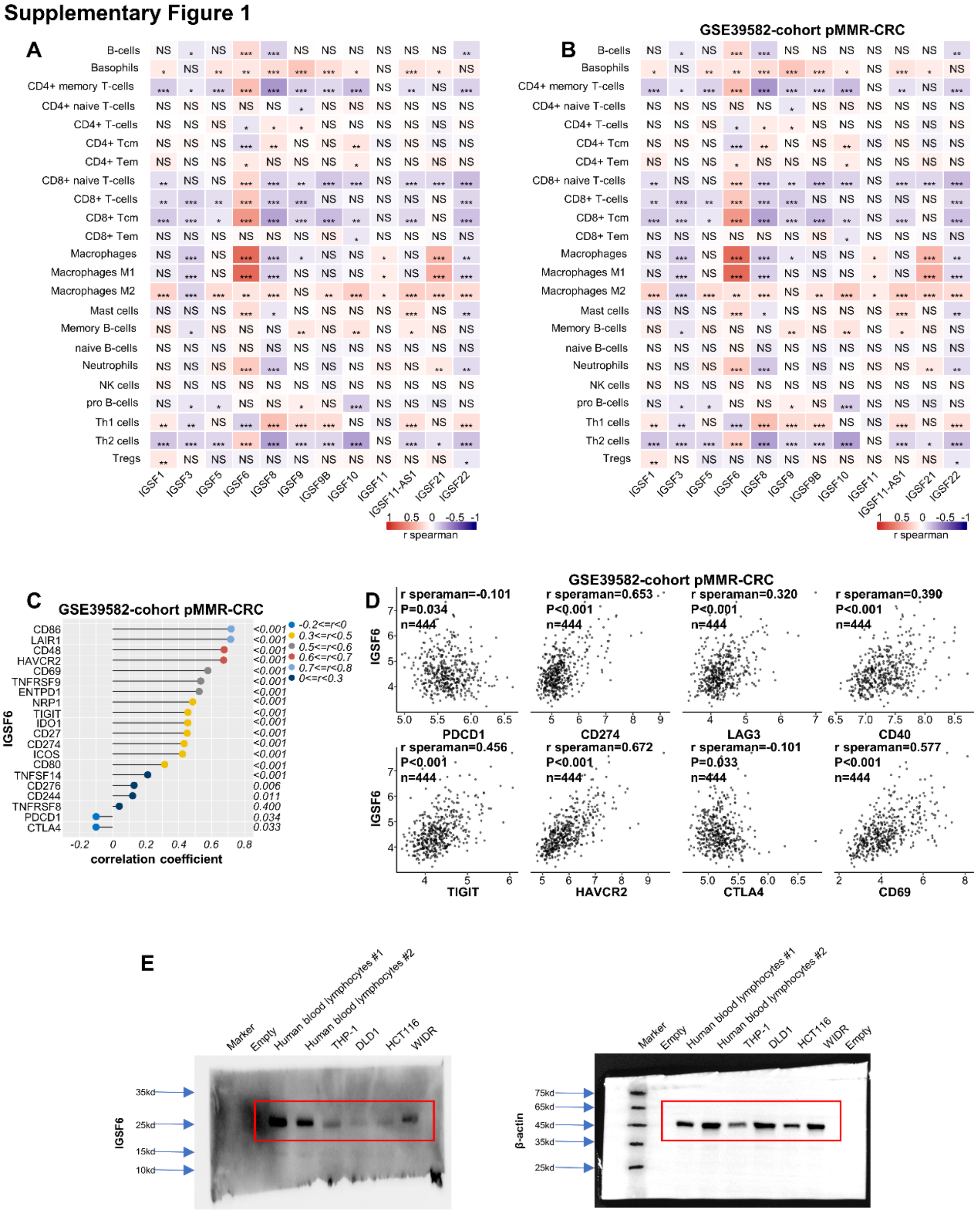

Supplementary figure 1. (A) Correlation between immune cell infiltration and IGSF in CRC patients from GSE39582 database, n=566. (B) Correlation between immune cell infiltration and IGSF in MMR-proficient CRC patients from GSE39582 database, n=444. (C, D) Correlation between immune check points and IGSF6 expression in MMR-proficient patients by Spearman's correlation coefficient, n=444. (E) The original blots of Figure 2F. Following the IGSF6 and β-actin datasheet, the membrane probed for IGSF6 was cropped at

approximately 45 kd and 10 kd; the membrane probed for β-actin was initially cropped around 90 kd and 15 kd, retaining sufficiently long lanes. We unanimously agree that the membrane clearly indicates the position of IGSF6 and β-actin.

Supplementary Figure 2

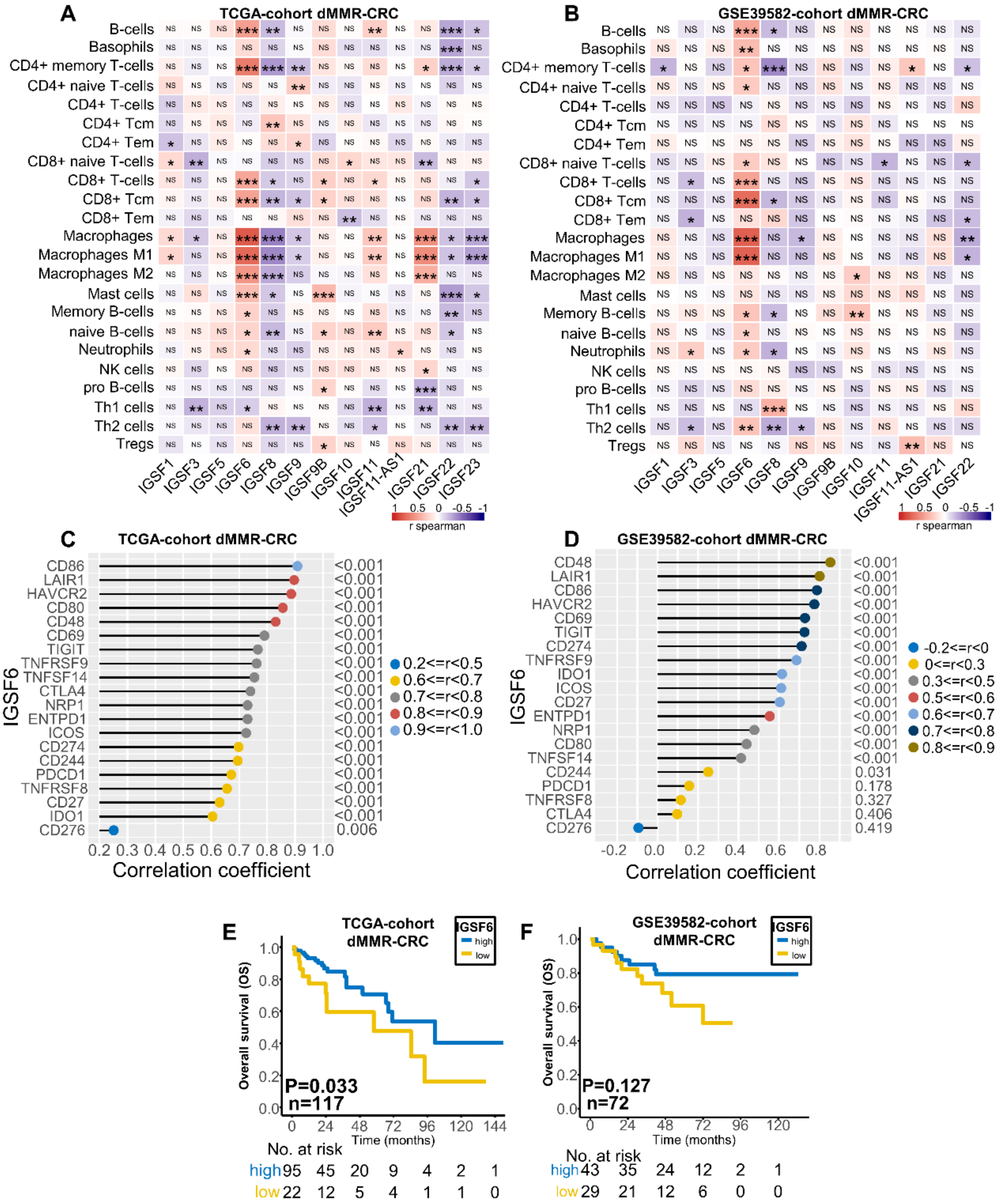

Supplementary Figure 2. (A, B) Correlation between immune cell infiltration and IGSF in MMR- deficient CRC patients from TCGA database, n=118 (A) and gse39582 database, n=75 (B). (C, D) Correlation between immune check points and IGSF6 expression in MMR- deficient patients by

Spearman's correlation coefficient, from TCGA database, n=118 (C) and gse39582 database, n=75 (D). (E, F) OS curve of patients with high IGSF6 and low IGSF6 group in TCGA database (E) and GSE39582 database (F).
